# Clinical characterization of neonatal and pediatric enteroviral infections: an Italian single center study

**DOI:** 10.1186/s13052-019-0689-8

**Published:** 2019-08-02

**Authors:** Alberto Berardi, Marcello Sandoni, Carlotta Toffoli, Alessandra Boncompagni, William Gennari, Maria Barbara Bergamini, Laura Lucaccioni, Lorenzo Iughetti

**Affiliations:** 1 Struttura Complessa di Neonatologia, Azienda Ospedaliero-Universitaria Policlinico, Via del Pozzo, 71, 41124 Modena, MO Italy; 2 Scuola di Specializzazione in Pediatria, Università di Modena e Reggio Emilia, Modena, Italy; 3 Struttura Complessa di Microbiologia e Virologia-Azienda Ospedaliero-Universitaria Policlinico, Modena, Italy; 4 Struttura Complessa di Pediatria, Azienda Ospedaliero-Universitaria Policlinico, Modena, Italy

**Keywords:** Enterovirus, Infection, Infant, Clinical findings, Outcome

## Abstract

**Background:**

Enteroviruses (EVs) are an important cause of illness, especially in neonates and young infants. Clinical and laboratory findings at different ages, brain imaging, and outcomes have been inadequately investigated.

**Methods:**

We retrospectively investigated EV infections occurring at an Italian tertiary care center during 2006–2017. Cases were confirmed with a positive polymerase chain reaction on blood or cerebrospinal fluid. Clinical and laboratory findings according to age at presentation were analyzed.

**Results:**

Among 61 cases of EV infection, 56 had meningitis, 4 had encephalitis, and 1 had unspecific febrile illness. Forty-seven cases (77.0%) presented at less than 1 year of age, and most were less than 90 days of age (*n* = 44). Presentation with fever (*p* < 0.01), higher median temperature (*p* < 0.01), and irritability (*p* < 0.01) were significantly more common among infants aged less than 90 days, who also had significantly higher peak temperatures during the course of the disease (*p* < 0.01). In contrast, gastrointestinal symptoms were more common in infants and children aged over 90 days (*p* = 0.02). Only 4 of 61 infections (6.5%) were severe and all affected younger infants (*p* < 0.01).

**Conclusions:**

We detail epidemiological, clinical, and laboratory findings in a cohort of 61 children. Infants aged less than 90 days have more severe disease; they are more likely to present with fever, higher median temperature, and irritability and less likely to develop gastrointestinal symptoms.

## Background

Enteroviruses (EVs) are small, single-stranded, positive sense RNA viruses belonging to the family *Picornaviridae*. Currently, more than 100 EV serotypes are known to infect humans and have been classified into 4 species (from A to D) [1]. EVs have a worldwide distribution and show seasonal patterns of incidence [2]. EVs are primarily transmitted from person to person via fecal-oral or respiratory routes. The primary replication sites are the epithelial cells of the oropharyngeal and intestinal mucosa. A viremia may lead to secondary infections of the central nervous system, myocardium, liver, and pleura [3]. Due to immaturity of the immune system, both the incidence and severity of EV infections are higher among younger infants. Risk factors in neonates include absence of neutralizing antibodies to the specific infecting serotype, maternal illness prior to or at delivery, prematurity, illness onset within the first few days of life, multi-organ disease, severe hepatitis, and infecting serotype [4]. Although infections in the pediatric population are mostly asymptomatic, clinical manifestations may range from minor febrile illnesses to severe, sometimes fatal conditions, including aseptic meningitis, encephalitis, acute flaccid paralysis, myocarditis, and acute hepatitis [5–8]. The polymerase chain reaction (PCR) represents the gold standard for the diagnosis of EV infections [9], and helps to reduce the length of stay and inappropriate antibiotic use [10–12].

Previous studies have reported the molecular epidemiology of EV infections [13–15], but data regarding the clinical presentation at different ages and their short-term outcomes have not been investigated in detail. Furthermore, there are no recommendations for managing neonates with confirmed EV infection, so that the decision to perform accurate investigations (such as MRI or EEG) is left to individual clinician. The aim of this study was to define the burden and short-term outcome of EV infections in a cohort of children admitted to an Italian tertiary care center. Furthermore, in order to define the clinical spectrum and to assess whether patterns of disease may vary according to age, we compared clinical and laboratory findings of cases aged less or more than 90 days.

## Methods

### Study design

This is a single center, retrospective cohort study of EV infections carried out at Azienda Ospedaliero-Universitaria Policlinico (Modena, Italy), a tertiary care center. This is a pediatric referral hospital for the whole city, with approximately 3500 live births/year, 400 neonatal and 1600 pediatric admissions/year. The laboratory database was searched for cases of positive EV-PCR in cerebrospinal fluid (CSF) or plasma samples of children admitted to the nursery, neonatal intensive care unit (NICU), and pediatric units between January 2006 and December 2017. Characteristics of each EV infection were recorded using a standardized form. Details included maternal history, sex, age at presentation, clinical symptoms, laboratory data, catecholamine support, antibiotic therapies, length of stay, imaging and outcomes. For neonates and infants, maternal data (mode of delivery, breastfeeding) were also recorded. The project was approved by the local ethical committee (No 4658/2017).

### Virological methods

Nucleic acids were extracted from 1 mL of CSF or plasma by using NucliSENS EasyMAG instrument (bioMérieux, Marcy l’Etoile, France) and submitted to amplification by Real Time Reverse Transcriptase Polymerase Chain Reaction for EVs. The sensitivity of the method was 10 copies/reaction. The specificity was 100% for negative samples whereas cross reactivity was absent (ELITechGroup, S.p.A, Turin, Italy). Both sensitivity and specificity were evaluated by external quality control as NEQAS and QCMD.

### Definitions

*Aseptic meningitis* was a clinical state indicating meningitis with a positive CSF EV-PCR (in absence of bacterial pathogens).

*Encephalitis* was aseptic meningitis associated with reduced consciousness (lasting more than 24 h) and at least 3 of the following: seizures, focal neurologic signs, raised CSF white blood cell (WBC) count (defined as ≥20/μL, 0–30 days of life; ≥15/μL, 30–60 days of life; or ≥ 5/μL, > 60 days of life) [16, 17], neuroimaging suggestive of encephalitis, and abnormal electroencephalography (EEG) consistent with encephalitis [18].

*Hepatic necrosis with coagulopathy* was associated with aspartate transaminase or alanine transaminase > 3 times the upper limit of normal and platelet count < 100,000/μL, plus an abnormal coagulation profile [19].

*Myocarditis* was a positive CSF and/or plasma EV-PCR plus an abnormal heart ultrasound study (ejection fraction < 50%, signs of congestive heart failure), or increased troponin I [20].

*Severe infection* included at least one of the following: encephalitis, mechanical ventilation, catecholamine support.

### Neurodevelopmental assessment

Neurodevelopmental assessment was performed in high-risk patients (neonates with documented brain lesions on magnetic resonance imaging [MRI] and/or severe infection) and consisted of serial clinical evaluations at 3, 6, 12, and 24 months of age. Neurological assessment was performed according to the Amiel-Tyson evaluation. Moreover, the Griffiths Mental Development Scale III [21] was performed to assess the rate of development at 6, 12, and 24 months.

### Statistical analysis

Analyses were performed using Medcalc version 9.3.0.0 for Windows. Continuous variables were expressed as mean ± SD or median and range; categorical data were expressed as numbers (percentages). Student’s t-test and Levene’s test for assessing homoscedasticity or the Mann-Whitney rank sum test were used to compare continuous variables, whereas the μ^2^ test or 2-tailed Fisher’s exact test was used to compare categorical variables between groups. All *p* values refer to 2-tailed tests of significance; *p* < 0.05 was considered significant.

## Results

### Characteristics of the population and epidemiological data

**Fig. 1:**
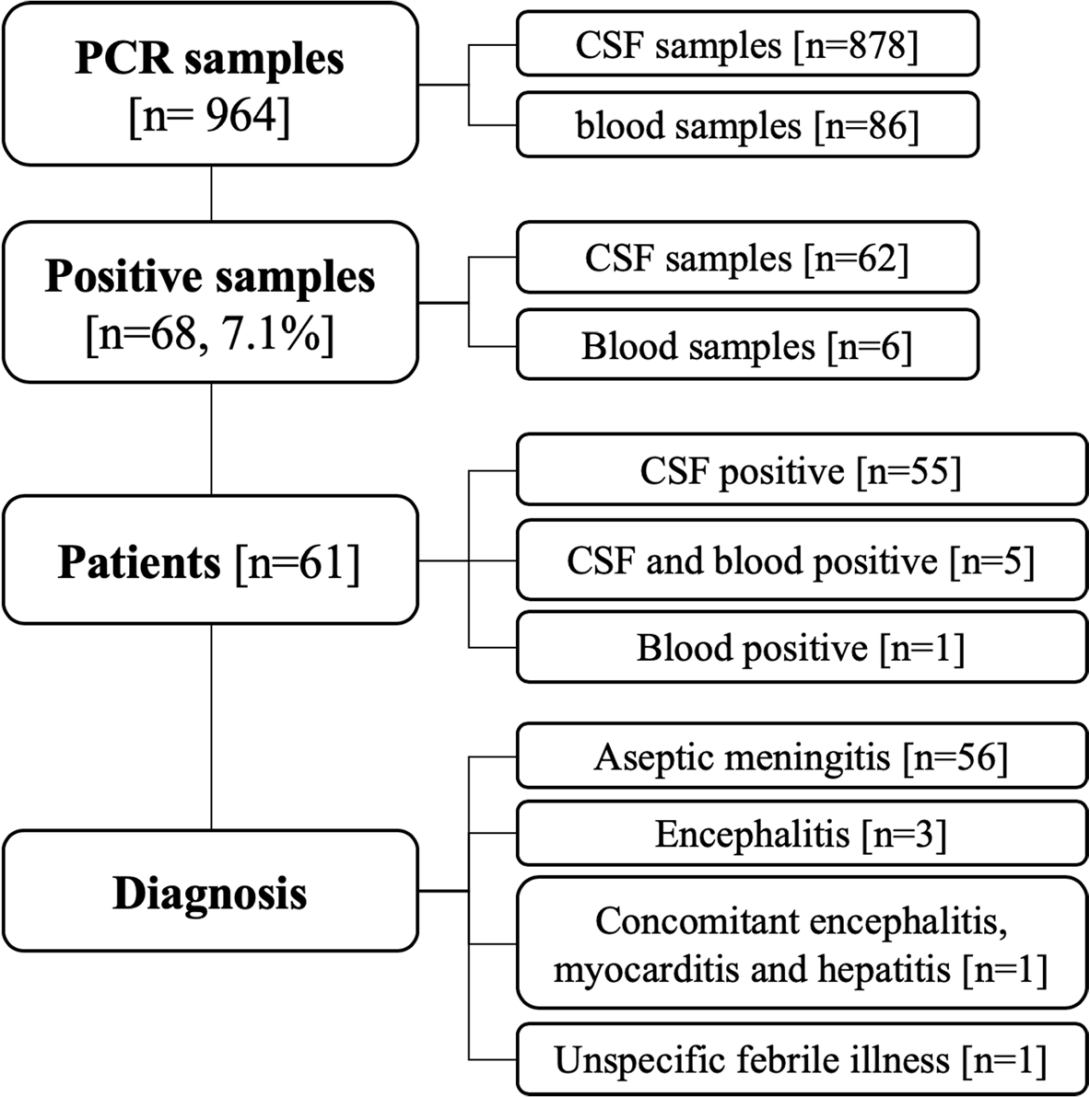
Virological samples, patients and diagnoses. CSF, cerebrospinal fluid; PCR, polymerase chain reaction

Figure 1 shows the number of positive samples in blood or CSF and the final diagnosis in the entire population. There were 68 EV-PCR-positive samples among 964 tested. Most samples were obtained from CSF. Among 878 CSF samples, 62 (7.1%) were EV positive.

**Fig. 2:**
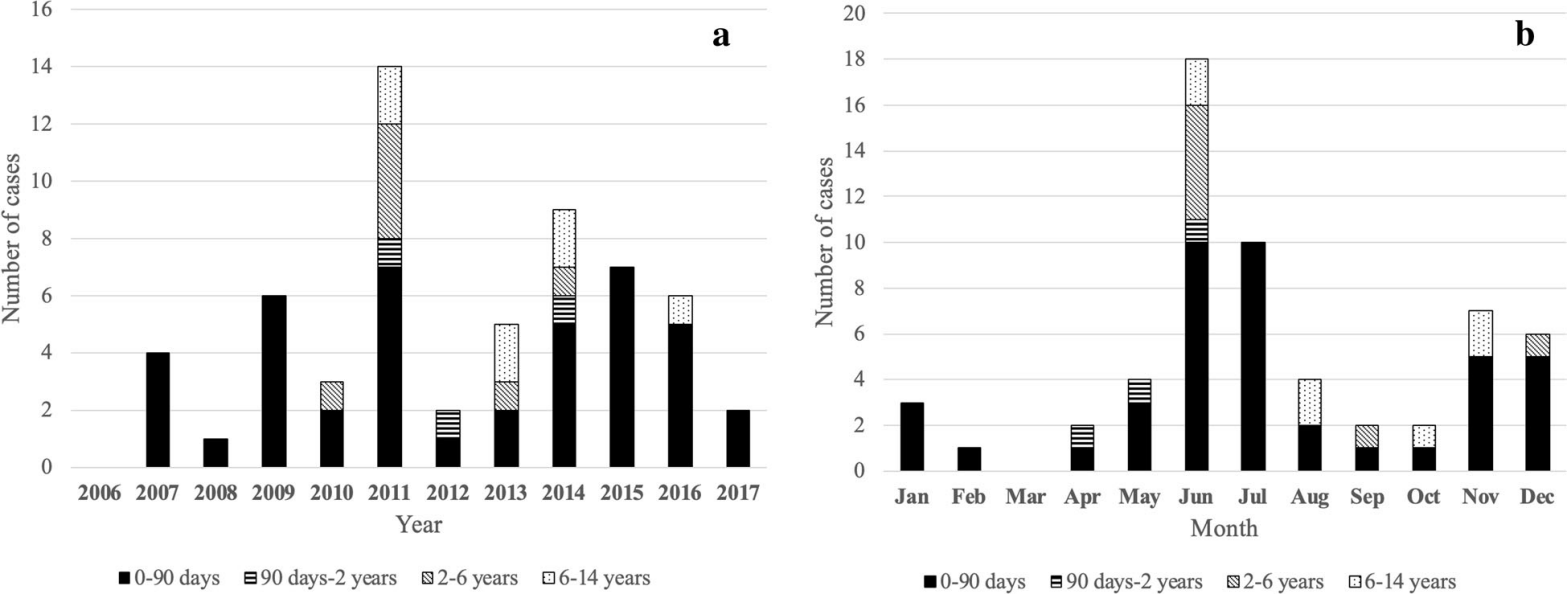
Yearly (**a**) and monthly (**b**) distribution of enteroviral infections according to age at presentation

Samples belonged to 61 children (boys, *n* = 35; girls, *n* = 26), of which 7 (11.5%) were admitted to the NICU, and 54 (88.5%) were admitted to the Pediatric Unit. Most cases had aseptic meningitis (cases aged less than 90 days: *n* = 39), a few had encephalitis (aged less than 90 days: *n* = 4). Lumbar puncture could not be performed in 1 infant aged less than 90 days and his illness was therefore classified as “unspecific febrile illness”. Age at presentation of EV infections ranged from 2 days to 9 years. Forty-seven of 61 (77.0%) presented within age 1 year (of which 44 were under 90 days). The remaining 14 (23.0%) presented after age 1 year. All 4 cases of severe EV infection were aged less than 2 weeks. Figure 2a details cases of EV infection during the 12-year study and Fig. 2b details month of presentation, according to age. There was a wide fluctuation in the annual number of cases during the study, and in some years no cases occurred among older children. Most cases had a seasonal pattern and presented during summer, with a peak in June–July.

### Mode of delivery and mode of feeding among infants aged 0 to 90 days

Most infants were born full-term (*n* = 37, 84.1%), after an uncomplicated pregnancy and delivery (*n* = 36, 81,8%). History of concomitant upper respiratory tract infection among family members was reported in 10 cases (22.7%). Median 5-min Apgar score was 10 (range 8–10). Seven (15.9%) were born preterm (median gestational age 35 weeks, range 31–36). Median birth weight was 3205 g (IQR 2870-3683) and median gestational age was 39 weeks (IQR 38–40). Most cases were exclusively breastfed (*n* = 23, 52.3%).

### Clinical characterization

Among infants aged 0 to 90 days, severe cases were more likely to be younger at presentation than non-severe cases (median age 6.2 vs 26.3 days, *p* = 0.003). Clinical findings at presentation and during the course of the disease are detailed in Table 1 according to age [21].

**Table 1:** Clinical findings of enteroviral infections in all age groups

Clinical features	Total (*n* = 61)	0–90 days (*n* = 44)	> 90 days (*n* = 17)	*p*
At presentation				
Fever, *n (%)*	42 (68.9)	36 (81.8)	6 (37.5)	< 0.01
Temperature, *median (IQR)*	38 (37.4–38.2)	38 (37.7–38.3)	37.4 (37.2–37.8)	< 0.01
Irritability, *n (%)*	29 (47.5)	26 (59.1)	3 (17.6)	< 0.01
Poor feeding, *n (%)*	18 (29.5)	14 (31.8)	4 (23.5)	0.74
Capillary refill time > 2″, *n (%)*	17 (27.9)	15 (34.1)	2 (11.8)	0.15
Tachycardia, *n* ^a^	23 (37.7)	14 (31.8)	9 (52.9)	0.22
Convulsions, *n (%)*	3 (4.9)	3 (6.8)	0	0.65
During the course of the disease				
Fever, *n (%)*	58 (95.1)	41 (93.2)	17 (100)	0.66
Duration (days), *median (IQR)*	2 (1–3)	2 (2–4)	2 (1–2)	0.05
Peak temperature (°C), *median (IQR)*	38.5 (38–39)	38.6 (38.2–39)	38.2 (38–38.5)	0.02
Gastrointestinal symptoms^b^, *n (%)*	27 (44.3)	13 (29.5)	14 (82.3)	< 0.01
Rash, *n (%)*	13 (21.3)	12 (27.3)	1 (5.9)	0.14

*NA* Not assessable

^a^ Normal values according to age reported in reference [22]

^b^ One or more episodes of diarrhea and/or vomiting

Presentation with fever, higher median temperature, and irritability were significantly more common among infants aged less than 90 days. During the course of the disease, the peak temperature was significantly higher among infants aged less than 90 days, whereas gastrointestinal symptoms were more common in the older population. Cutaneous rash (characterized by small, erythematous macular or maculopapular lesions), tachycardia and capillary refill time > 2 s did not differ between the 2 age groups.

### Laboratory tests, instrumental data

Blood tests and CSF parameters according to age [16, 17, 23] are detailed in Table 2.

**Table 2:** Laboratory tests at presentation

	Total (*n* = 61)	NA	0–90 days (*n* = 44)	NA	> 90 days (*n* = 17)	NA	*p*
Blood testing							
WBC count, (/μL), *median (IQR)* ^a^	10,550 (6900-13,225)	0	9110 (6508-11,568)	0	12,010 (10,578-15,680)	0	NC
Raised WBC count, *n (%)*	10 (16.4)	0	1 (2.3)	0	9 (52.9)	0	< 0.01
Low WBC count, *n (%)*	16 (26.2)	0	16 (36.4)	0	0	0	0.01
CRP (mg/dL), *median (IQR)* ^b^	0.81 (0.10–1.40)	6	0.81 (0.30–1.38)	6	0.91 (0.10–2.85)	0	0.60
CRP > 1 mg/dL, *n (%)*	24 (43.6)		16 (42.1)		8 (47.1)		0.60
CRP > 5 mg/dL, *n (%)* ^c^	4 (7.3)		2 (5.3)		2 (11.8)		0.66
PLT (thous/μL), *median (IQR)* ^a^	305 (250–380)	0	309 (244–392)	0	293 (267–345)	0	0.65
Low PLT count, *n (%)*	1 (1.6)	0	1 (2.3)	0	0	0	0.62
ALT (mg/dL), *median (IQR)* ^a^	22 (15–30)	2	25 (19–34)	2	14 (9–17)	0	NC
Raised ALT levels, *n (%)*	7 (11.9)	2	7 (16.7)	2	0	0	0.17
Total bilirubin (mg/dL), *median (IQR)*	3.56 (1.27–5.83)	32	3.56 (1.27–5.83)	15	–	17	NC
CSF parameters^d^							
CSF cell count (/μL), *median (IQR, range)*	39 (4–250, 0–1864)	11	8 (2–185, 0–1864)	9	108 (44–339, 12–1256)	2	NC
CSF pleocytosis, *n (%)*	30 (60.0)		16 (45.7)		15 (100)		< 0.01
CSF protein (mg/dL), *median (IQR)*	45.5 (33–68)	11	58.5 (39–101)	9	24 (15–42.8)	2	NC
Raised CSF protein, *n (%)*	15 (30.0)		13 (37.1)		2 (13.3)		0.18
CSF glucose (mg/dL), *median (IQR)*	46 (40–51)	5	43.5 (39–46.5)	4	58.5 (48–71)	1	NC
Low CSF glucose, *n (%)*	4 (7.1)		3 (7.5)		1 (6.3)		0.68

*CRP* C-reactive Protein, *CSF* Cerebrospinal fluid, *Hb* Haemoglobin, *NA* Number of cases for which data were not available; *NC* Not comparable; *PLT* Platelets; *WBC* White blood cell

^a^ Normal values according to age reported in reference [23]

^b^ Children with concomitant urinary tract infection were excluded from the analysis

^c^ Peak CRP > 5 mg/dL during the course of the disease was present in 8 (18.2%) infants aged 0 to 90 days and 3 (17.6%) aged more than 90 days (11, 18.0% of the total population);

^d^ Traumatic lumbar punctures were excluded from the comparison of CSF cell count and protein levels. Pleocytosis was defined as ≥ 20/μL, 0–30 days of life; ≥ 15/μL, 30–60 days of life; or ≥ 5/μL, > 60 days of life. Raised CSF protein level was > 90 mg/dL, 0–30 days of life; and > 45 mg/dL, > 30 days of life. Low CSF glucose was < 37 mg/dL, 0–30 days of life; and < 40, > 30 days of life [16, 17].

CSF pleocytosis (with wide variation in cell count) and increased WBC count were significantly more common among children older than 90 days, whereas a low WBC count was more common among younger patients.

At presentation, C-reactive protein levels were mostly within a normal range or mildly raised, but a few cases (< 10% of children) had levels over 5 mg/dL. During the course of the disease, C-reactive protein levels increased to over 5 mg/dL in 18% of the entire population, without significant differences between the 2 age groups (data not shown).

Bilirubin levels were only assessed in younger infants. Most levels were within a normal range, but levels were markedly raised in 2 cases with severe infection (22 and 17 mg/dl respectively).

Platelet count and alanine transaminase levels were within normal range in most children. A few cases had elevated CSF protein or low CSF glucose levels.

Table 3 details heart and brain investigations. Most infants younger than 90 days underwent ultrasound study; brain MRI or computed tomography was only performed in a few children. Brain lesions were documented in 3 of 4 severely infected newborns who underwent MRI.

**Table 3:** Heart and brain study of enteroviral infections in all age groups

	0–90 days (*n* = 44)		> 90 days (*n* = 17)		Total (*n* = 61)	
	Performed, *n*	Abnormal, *n*	Performed, *n*	Abnormal, *n*	Performed, *n*	Abnormal, *n*
ECG recording*, n (%)*	44	1 (2.3)^a^	17	0	61	1 (1.6)
Heart US study*, n (%)*	30	3 (10) ^b^	7	0	37	3 (8.1)
Brain US study*, n (%)*	27	3 (11.1) ^c^	–		27	3 (11.1)
Brain MRI*, n (%)*	8	3 (37.5) ^d^	1	0	9	3 (33.3)
Brain CT*, n (%)*	–		7	0	7	0
EEG recording*, n (%)*	10	3 (30)	8	2 (25)	18	5 ^e^ (27.8)

*CT* Computed tomography, *ECG* Electrocardiogram, *EEG* Electroencephalography, *MRI* Magnetic resonance imaging, *US* Ultrasound

^a^ Abnormal repolarization

^b^ Low ejection fraction (*n* = 1), hyperechoic interventricular septum (*n* = 1), pericardial effusions (*n* = 1)

^c^ Hyperechoic spots in the white matter and basal ganglia (*n* = 2), thinned brain ventricle (*n* = 1)

^d^ Hyper-intense signal areas of brain white matter in T1 or T2-weighted images

^e^ Anterior slow dysrhythmia (*n* = 4), status epilepticus, (*n* = 1), suppression burst patterns (*n* = 1) excess of theta rhythm (*n* = 1)

### Therapies and outcome

Median duration of antibiotic therapy was calculated by excluding all children with concomitant bacterial urinary tract infection (*n* = 6). Median was 6 days for infants aged less than 90 days (interquartile range [IQR] 5.5–8.0 range 3–13 days; μ 5 days, *n* = 27) and 7 days for infants aged more than 90 days (IQR 5.0–7.0, range 3–9 days; ≥ 5 days, *n* = 10). A few infants were given antiepileptic drugs (*n* = 3), underwent mechanical ventilation (*n* = 2), or catecholamine support (*n* = 1). One newborn underwent extracorporeal membrane oxygenation. No deaths were recorded. Median length of stay was 7 days (IQR 6–10). Neurodevelopmental outcome at age 2 years in the 4 severely infected newborns with documented MRI brain lesions was within normal range, but 1 developed epilepsy at age 6 years.

## Discussion

The current study provides detailed information regarding clinical characteristics of neonatal and pediatric EV infections from an Italian University-Teaching Hospital over a 12-year period. No case fatalities were recorded. In the past, EV infections have been an important cause of mortality and morbidity in the pediatric age group, with most symptomatic and severe cases occurring during the first months of life. Neonatal mortality rates vary widely (from 0 to 83%) in the literature, according to the study population (children, neonates, severe infections) [7, 24, 25], but lower mortality rates (0–10%) have recently been reported [20, 26–29]. Myocarditis and hepatitis with coagulopathy are associated with greater risks of death [19, 30].

In the present study, the total number of cases of EV infection showed large fluctuations, and in some years, no cases were recorded among older children. Consistent with previous studies, we found that most cases had a seasonal pattern with a peak during summer [1], and most cases presented under age 90 days in full-term newborns who had an uncomplicated pregnancy and delivery [8]. A maternal history of viral illness is frequently (59–68%) reported in previous studies [25]. Although we found that concomitant upper respiratory tract infection among family members was uncommon (22%), this information may have been biased by the retrospective design of our study.

Most newborns had aseptic meningitis and a few had encephalitis. This finding is consistent with large epidemiological studies that describe EV as a primary cause of viral meningitis (48–95%) in high-income settings [31, 32]. In the current study, approximately 7% of CSF samples collected because of clinical suspicion were confirmed EV positive.

The earlier the age at presentation, the greater the severity of disease; in fact, all severe cases occurred within age 2 weeks. This finding is consistent with previous literature [7, 8]. Furthermore, we compared clinical findings between infants younger and older than 90 days. Fever was common in both populations, and seizures and cutaneous rashes were prominent among younger children, whereas gastrointestinal symptoms and nuchal rigidity were predominant among older ones.

Although limits of CSF pleocytosis (defined according to age) were different, infants aged less than 90 days were more likely to present with a lower WBC count and less likely to present with CSF pleocytosis. Consistent with previous studies [30], total bilirubin levels were significantly higher in more severe EV infections, although this finding would be related to the higher bilirubin levels at younger ages rather than the severity of the disease. Only 5% of children had seizures requiring antiepileptic treatment. All children without concomitant bacterial infection underwent antibiotic therapy that lasted 5 or more days in approximately 2/3 of cases. C-reactive protein levels increased in only a few cases and could not account for the prolonged antibiotic treatments, since more recent literature suggests discontinuing therapy when EV infection is confirmed [33].

This study has a number of limitations. First, the retrospective design may have biased some information. Furthermore, we could not associate clinical findings with molecular / genomic data. Indeed, the medical literature reports variations in the clinical presentation and CSF findings with different EV subtypes. Furthermore, the diagnosis of EV infection did not include PCR on stool and respiratory specimens (as reported in more recent studies by others) [34]. MRI represents the most sensitive imaging technique in patients with severe infection and focal neurologic signs, and is advisable in the presence of profound lethargy or neurologic deterioration [35]. In the current study, most but not all children with abnormal neurological signs (i.e. hypotonia, abnormal EEG) underwent MRI; therefore, encephalitis and severe disease may have been to some extent underestimated. Finally, only a few children (mostly those with severe infection, convulsions, or myocarditis) underwent neurodevelopmental follow-up. Early studies reported white matter abnormalities and long-term impairment (such as neurodevelopmental delay, cerebral palsy, and epilepsy), in surviving neonates after NICU admission due to EV infection. During the 80s, Wilfert [36] showed that receptive language function was significantly diminished in 9 children with EV infection compared to that in 9 controls. More recently, Verboon-Maciolek [35] studied 6 infants aged less than 60 days with meningoencephalitis presenting with seizure or hypotonia; 2 of 6 developed cerebral palsy and epilepsy. Among 17 confirmed EV encephalitis cases, Pillai [37] reported abnormal outcomes in 8 cases, of which 3 developed epilepsy. However, there is a lack of large studies detailing the neurodevelopmental outcome of EV infections.

## Conclusions

We reviewed cases of EV infections from an Italian pediatric cohort and we confirmed that EV represent a relevant cause of illness and CNS infection. Clinical characteristics may vary according to age of presentation, but more severe infections occur among infants aged less than 90 days. Our findings suggest that neurodevelopmental follow-up should be planned after CNS EV infections.

## Data Availability

The datasets used and/or analysed during the current study available from the corresponding author on reasonable request.

## Abbreviations

CNS: Central nervous system
CSF: Cerebrospinal fluid
EEG: Electroencephalogram
EV: Enterovirus
IQR: Interquartile range
MRI: Magnetic resonance imaging
NICU: Neonatal intensive care unit
PCR: Polymerase chain reaction
WBC: White blood cell
